# The Aging Population Faces Increased Risk for Musculoskeletal Pathologies: The Problematic Atlas-Axis Instability

**DOI:** 10.7759/cureus.15068

**Published:** 2021-05-17

**Authors:** Antonia Nituleasa, Elizabeth D Liu, Ryan F Amidon, Christ Ordookhanian, Paul Kaloostian

**Affiliations:** 1 Cell, Molecular, and Developmental Biology, University of California Riverside, Riverside, USA; 2 Biochemistry, University of California Riverside, Riverside, USA; 3 Medicine, Medical College of Wisconsin, Milwaukee, USA; 4 Medicine, University of California Riverside, Riverside, USA; 5 Neurological Surgery, Riverside Community Hospital, Riverside, USA; 6 Neurological Surgery, Paul Kaloostian M.D. Inc., Riverside, USA

**Keywords:** spinal cord injury, atlantoaxial instability, end of life care, quadriplegia, pannus, pars fracture, elderly population

## Abstract

Spinal cord injury (SCI), particularly of the traumatic variety, is a relatively common condition that disproportionately affects the elderly. Cases of SCI with nontraumatic etiologies in the geriatric population have increased over the last 20 years, however. Pannus formation, resulting from chronic inflammation of the spine, is one such etiology that may progress to SCI and potentially result in rapid neurological degeneration. Here we describe a case of an elderly woman who presented with a sudden onset of quadriplegia without a history of trauma. Radiography revealed upper cervical instability and fracture due to the presence of a large erosive pannus formation. Unfortunately, in the context of severe SCI, the reversibility of neurological decline is not always guaranteed. Additionally, surgical intervention is not always appropriate, especially among the elderly population, where medical management and end-of-life care are more often delivered.

## Introduction

Spinal cord injuries (SCIs) represent a growing public health obstacle. Despite countless advancements in medicine and technology, they often result in re-hospitalization and reduced life expectancies due to consequences affecting other organ systems [1,2]. One epidemiological study found that SCI disproportionately affects the elderly population (individuals of 65 years and older), with estimates of 47.5 cases per million people in comparison to a general population incidence of 18.8 cases per million people [2]. The same study found an alarming upward trend in the number of SCI cases in the elderly, increasing from a prevalence of 4.2% to 15.4% over the course of 20 years. This trend, coupled with the aging population, magnifies the importance of appropriately managing the abundance of possible manifestations of SCI in the elderly.

A higher mortality rate is commonly reported in the elderly population with SCIs in comparison to the general population with SCIs, as evident from seven-year survival rates dropping from 86.7% to 22.7% in patients over 50 years old [2]. Mortality rates in elderly individuals range from 24% to 26% for SCIs and mortality rates double in those with quadriplegia or paraplegia [1]. Mortality rates are highest in the first year following the injury, especially for those with neurological sequelae [3]. SCI-related reduction of life expectancy is linked to cardiac events, respiratory failure, and septicemia [1-3].

Pannus formations, which constitute one nontraumatic etiology of SCI, are inflammatory granulation or fibrovascular tissues that affect the body’s synovial joints. Electron microscopic studies suggest that synovial derived fibroblast-like cells travel over a cartilage surface, creating the abnormal layer known as pannus. Cells gradually move into the pannus, creating fibrous tissue. Additionally, cells within the pannus formation produce proteinases that destroy the cartilage, resulting in joint instability [4]. Erosive pannus can precede bone destruction; as a result, erosive pannus at the cervical vertebra one and two (C1-C2) induces loss of ligamentous support, cumulating in atlantoaxial instability [5].

Most literature describes cervical pannus appearing in the upper cervical spine segments and often results from rheumatoid arthritis or trauma. Less commonly, it may arise spontaneously or from atypical etiologies including spontaneous cervical epidural hematoma and chronic odontoid fractures [6]. Joaquim et al. explained that when odontoid fractures arise in the atlantoaxial region (C1-C2), neurological symptoms are rare due to a relatively large spinal canal [7]. If pathologies of the cervical spine are left untreated or undiagnosed, however, significant neurological morbidity can develop [8].

In this case, we highlight the potential for pannus in the atlantoaxial region to induce severe cervical SCI leading to rapid neurological decline and poor prognosis. An elderly woman presented with sudden quadriplegia, which was later revealed to be a result of atlantoaxial instability and fracture from an erosive pannus formation. Given the severity of the SCI, a lack of neurological improvement from medical management, and a minimal chance of recovery and avoiding complications from surgery, the patient’s family opted for hospice comfort care. In light of the increasing prevalence of SCI and an aging population, broadening our understanding of the many possible manifestations of SCI, and cervical pannus, in particular, is becoming ever more crucial to improving outcomes in the geriatric population.

## Case presentation

**Figure 1:**
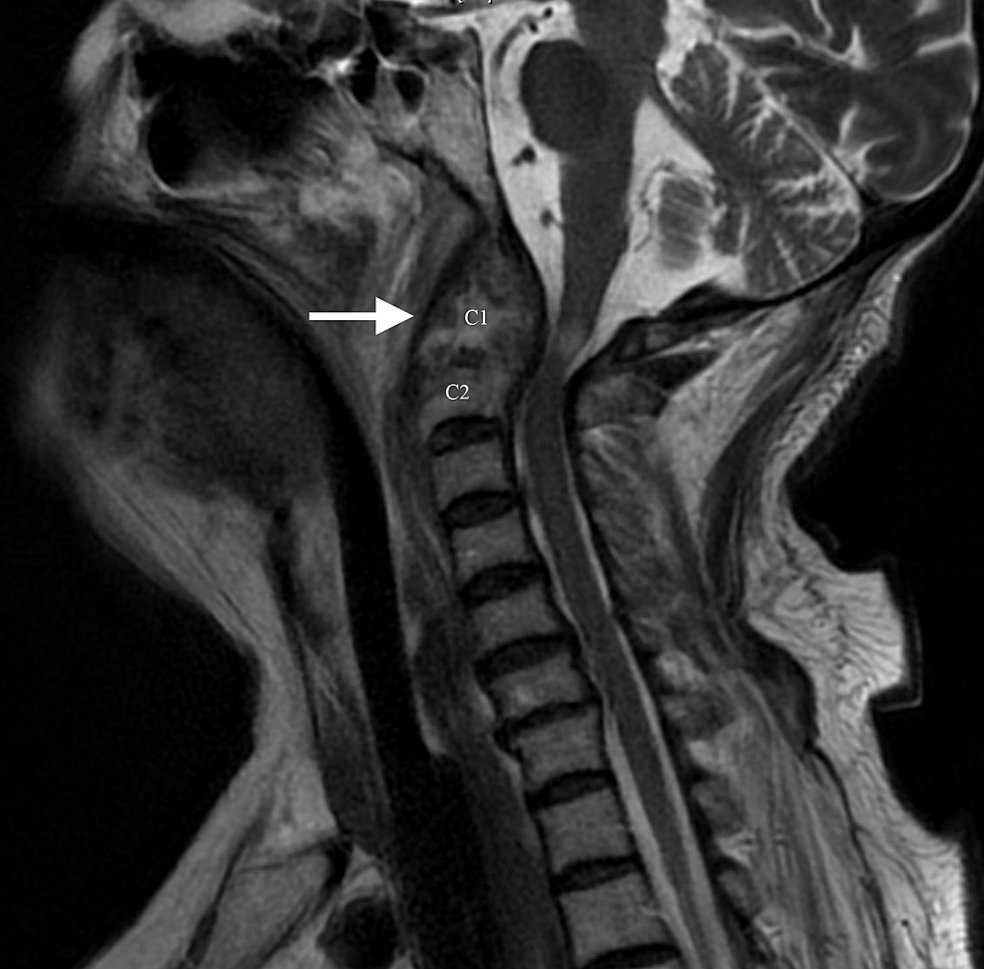
MRI cervical spine reveals pannus formation (arrow) at C1-C2 region. MRI: magnetic resonance imaging C1: cervical vertebra one C2: cervical vertebra two

An 80-year-old Caucasian woman presented to the hospital as a transfer with acute onset of quadriplegia coupled with ventilator dependence while being cared for at a nursing home. There was no evidence of any trauma. The quadriplegic development was reported to have been spontaneous in nature. Magnetic resonance imaging (MRI) of the cervical spine demonstrated a cervical vertebra one and two (C1-C2) large pannus formation (arrow) with spinal cord signal changes and severe spinal stenosis (Figure 1).

**Figure 2:**
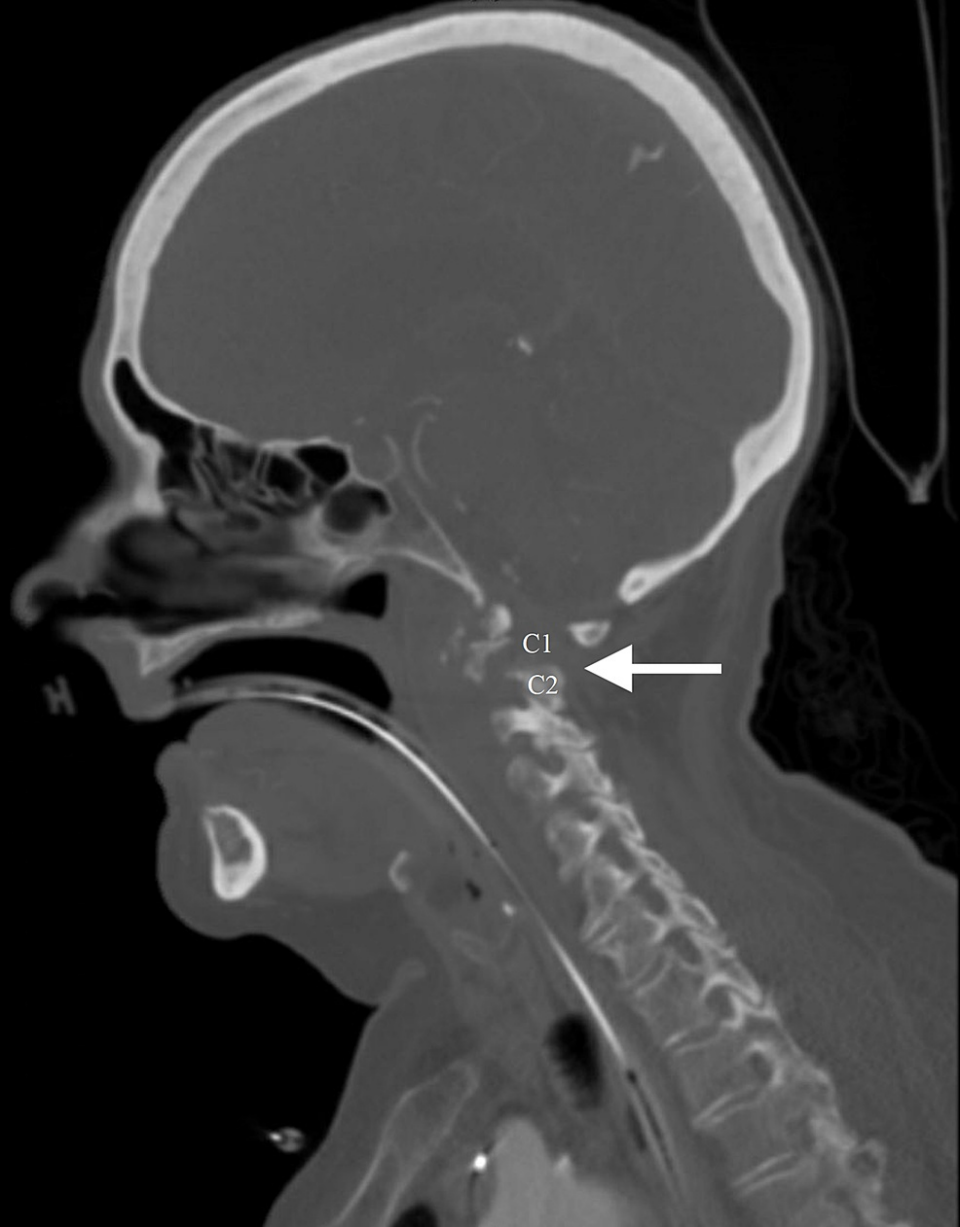
Cervical spine CT scan reveals C1-C2 degeneration with loss of bone integrity (arrow). CT: computed tomography C1: cervical vertebra one C2: cervical vertebra two

A computed tomography (CT) scan of the cervical spine showed a pars fracture at C2 with large erosive pannus (arrow) that eroded through C2 and C1 (Figure 2).

The patient was monitored in the neurological intensive care unit (Neuro-ICU) where mean arterial pressure (MAP) was pushed above 85 millimeters of mercury (mmHg) for seven days. Steroids (dexamethasone tapered down 4 milligrams (mg) every six hours from initial dose to 0 mg over one week) were administered. Given the severe SCI and no improvement in neurological condition, as well as a very low chance of any meaningful recovery from a surgical procedure, the family decided on hospice comfort care.

## Discussion

SCI is an umbrella term, encompassing numerous pathologies and presentations with varying etiologies. While 96% of all SCI cases result from trauma, nontraumatic SCIs are more prevalent in the elderly population and can be observed resulting from tumors, vascular disease, degenerative disease, inflammation, and other etiologies [2,9]. It is worth mentioning that natural, age-related physiological changes lead to a higher likelihood of cervical fractures and SCIs. The elderly population is more likely to experience diseases affecting bone integrity, including osteoporosis and osteopenia, placing them at higher risk of injury [2]. Degenerative changes of the spine are seen in 90% of men over 50 years old and 90% of women over 60 years old, which may lead to spondylosis and increase the prospect of SCIs, fractures, and spinal stenosis [2,9]. One potential nontraumatic etiology of SCI is pannus, an accumulation of granulation tissue leading to subsequent erosion of subchondral bone and resultant decrease of bone strength.

Following an SCI, the respiratory system is particularly impacted and respiratory failure remains a leading cause of death in SCI patients [10]. Cervical injuries commonly lead to dysfunctional respiratory muscle activity; subsequently, injuries above the cervical vertebra three (C3) level may affect the diaphragm muscles via disruption of the phrenic nerve, necessitating ventilator dependence [11]. Respiratory sequelae, including dyspnea, reduced lung and chest wall compliance, decreased lung volume, and diminished response to hypercapnia, have all been observed in patients with quadriplegia [11,12]. Such pulmonary changes lead to impaired inspiration and expiration. Thus, respiratory support may become necessary.

Since 2015, nearly 60% of individuals with SCI will experience neurological sequelae resulting in either complete or incomplete quadriplegia [3]. Quadriplegia, also known as tetraplegia, is the complete or partial paralysis of all four limbs and torso. This impairment or loss of function is attributed to damage of the cervical spinal cord. It can be categorized based on the damaged region, either as low quadriplegia (affecting C5-C8) or high quadriplegia (C1-C4), or based on the degree of movement and feeling [3]. Our patient experienced an acute onset of quadriplegia as a result of C1-C2 erosive pannus and subsequential atlantoaxial instability.

Without a history of any trauma, our patient's development of quadriplegia was deemed to be spontaneous in nature. Magnetic resonance imaging (MRI) revealed a large C1-C2 pannus formation. Such pannus formations without a history of rheumatoid arthritis or trauma are atypical; however, odontoid fractures and atlantoaxial instability have been implicated as likely sources of the invasive tissue [6,10]. Severe C1-C2 pannus can progress to vertebral erosion and compromise the transverse ligament of the atlas, promoting atlantoaxial instability [12]. CT imaging confirmed the patient had spondylolysis at the C2 vertebrae as well as degraded C1-C2 vertebrae resulting from a large, erosive pannus. Unlike other vertebrae, C1 and C2 are especially susceptible to subluxation [12]. Combined with compromised ligament integrity, increased dislocation susceptibility can lead to a higher risk of fracture in the odontoid processes and cause an increased risk of spinal cord compression [5,12]. Pannus-induced spinal cord compressions result in further spinal injury and other neurological sequelae. Given the localization of the pannus to the synovium of C1-C2, MRI unsurprisingly demonstrated evidence of spinal cord changes and severe spinal stenosis. Patients with spinal stenosis are at risk for the development of myelopathy and any reduction of the space available for the spinal cord to lesser than 14 mm serves as a reliable predictor of the likelihood and severity of paralysis [13].

Operative treatments in symptomatic adults are generally primary methods of treatment; however, surgical options were not pursued by our patient [13]. Following recommended guidelines, our patient’s MAP was maintained above 85 millimeters of mercury (mmHg) for seven days while in the neurological intensive care unit (Neuro-ICU). Maintaining MAP above 85 mmHg reduces the likelihood of spinal cord ischemia and has been observed to increase possibilities of neurological improvements [14]. For non-operative management of inflammation, the patient received steroids (dexamethasone). Corticosteroids, particularly dexamethasone, have long been established as part of SCI treatment plans for their anti-inflammatory effects and their role in facilitating neurological improvement [15].

Surgical treatments can be effective, but rates of complications, such as vertebral artery injury, cerebrospinal fluid leakage, and death, vary depending on operative technique with occurrences ranging from 0% to 9.4% [13]. In geriatric patients, preexisting comorbidities must be taken into account when determining a treatment course and palliative care may be an appropriate consideration [1]. Less than 1% of those with SCI will experience complete recovery of neurological symptoms at the time of hospital discharge and about 30% of patients with SCI will be re-hospitalized one or more times in any given year [3]. Although there is no significant difference between the neurological recovery between the older and younger populations, there is a notable divergence when examining functional recovery as the older population seldom experiences positive functional outcomes [2]. The goal of the medical management for our patient was to prevent conditions from worsening. Surgery was contraindicated due to the patient’s advanced age. Unfortunately, achieving neurological recovery was no longer possible. Accordingly, the patient’s family decided to place the patient in hospice comfort care.

## Conclusions

While frequently encountered in medicine, severe SCIs in the geriatric population have historically represented difficult pathologies to treat. Current patient status, the presence of comorbidities, degree of SCI severity, and etiology of SCI are among the many factors that steer medical decision-making. Surgical intervention is frequently contraindicated in elderly patients. Consequently, long-term rehabilitation or end-of-life care is pursued. In this case, we discuss the development of upper cervical erosive pannus in an elderly patient leading to spontaneous quadriplegia by means of severe SCI. Surgery held a poor prognosis due to the patient's age and condition. Therefore, medical management was delivered. The patient's family opted for hospice comfort care, prioritizing quality of life when the length of life and neurological restoration could no longer be pursued. This case aims to highlight the potential for erosive pannus formations to produce rapid and irreversible neurological decline.
